# Overt and Covert Speech-State Classification from Tripolar Concentric Ring Electrode EEG: A 4 mm and 10 mm Configuration Study

**DOI:** 10.3390/s26206587

**Published:** 2026-10-17

**Authors:** Paras Qadir Memon, Charles W. Anderson, Shoaib Memon

**Affiliations:** 1 Department of Computer Science, Colorado State University, Fort Collins, CO 80523, USA; chuck.anderson@colostate.edu; 2 Department of Computer and Information Sciences, University of Arkansas at Little Rock, Little Rock, AR 72204, USA; smemon@ualr.edu

**Keywords:** brain–computer interface, electroencephalography, tripolar concentric ring electrode, TCRE, overt speech, covert speech, EEG classification, surface Laplacian, speech-state classification

## Abstract

Electroencephalography (EEG) speech-state classification is a noninvasive method to identify the brain’s overt and covert behavior. The EEG achieved through the scalp is contaminated by artifacts and volume conduction and suffers from poor spatial resolution. We present the first application of small 4 mm tripolar concentric ring electrodes (TCREs) to record the overt and covert EEG signals. In addition, EEG signals were recorded for overt and covert behavior using a 10 mm TCRE as a reference. Data were recorded from 16 participants (4 mm, n=8; 10 mm, n=8). Each participant completed 200 picture-naming trials (100 overt and 100 covert). The analysis includes task-to-baseline root mean square ratios, power spectral density, time frequency representation analysis and participant-wise overt and covert classification using linear discriminant analysis, support vector machines, and EEGNet. The overt speech produced higher task-to-baseline ratios than covert speech. In addition, EEGNet achieved the highest mean accuracy (84.06±15.57%), followed by SVM (81.56±14.23%) and LDA (76.56±15.30%).

## 1. Introduction

Brain Computer Interfaces (BCIs) provide an alternative communication method for individuals with severe motor or speech-related disabilities [1,2]. A number of noninvasive modalities are available; among those, electroencephalography (EEG) is widely used due to its portability, low cost, and high temporal resolution [1,3]. EEG has been used to decode large body parts such as elbow, wrist, and leg movements [4,5,6]. However, EEG suffers from poor spatial resolution and volume conduction [7], as well as artifact contamination problems [8]. Therefore, there is a need to improve EEG recordings in order to achieve better local signal selectivity [9]. One approach to improve the spatial selectivity of EEG recordings is the use of tripolar concentric ring electrodes (TCREs). Besio et al. [9] developed a TCRE with a 10 mm outer ring diameter to calculate the surface Laplacian on the hardware to improve the spatial resolution of the EEG signals [10]. Unlike a conventional Laplacian approach, which requires neighboring electrodes, TCRE calculates the local surface Laplacian using the central disc, middle ring, and outer ring of the same electrodes. The TCRE-based surface Laplacian can be expressed as:

(1) $$SL_{\mathrm{TCRE}}=\frac{16\left(V_{m}-V_{d}\right)-\left(V_{o}-V_{d}\right)}{3R^{2}},$$

where $V_{d}$, $V_{m}$, and $V_{o}$ denote the potentials from the central disc, middle ring, and the outer ring, respectively. *R* is the radius of the middle ring. This hardware-based Laplacian can improve spatial selectivity, reduce sensitivity to distant sources, and contribute toward solving the reference electrode problem. Previous studies have reported improved spatial resolution, enhanced signal-to-noise ratio, and reduced artifact contamination when using TCREs compared with conventional EEG recordings [10,11,12]. A small TCRE with a 4 mm outer ring diameter was developed by Walter Besio at the Neural Rehabilitation Engineering Laboratory at the University of Rhode Island. The 4 mm TCRE has not previously been evaluated for speech or other EEG applications. In this work, we present the first evaluation of the 4 mm TCRE for overt and covert speech state classification. The 10 mm TCRE is used as a reference configuration. In speech state classification: overt speech refers to speech spoken aloud, whereas covert speech refers to internally imagined speech without vocalization [13,14,15]. A recent review has surveyed EEG-based imagined speech decoding, which addressed the challenges of inter-subject variability and generalizability [16]. Distinguishing these speech states from brain signals is challenging because speech-related neural activity is weak [15], varies across participants [17], and is often contaminated by artifacts from articulation and facial movement during overt speech [8,18].

**Figure 1:**
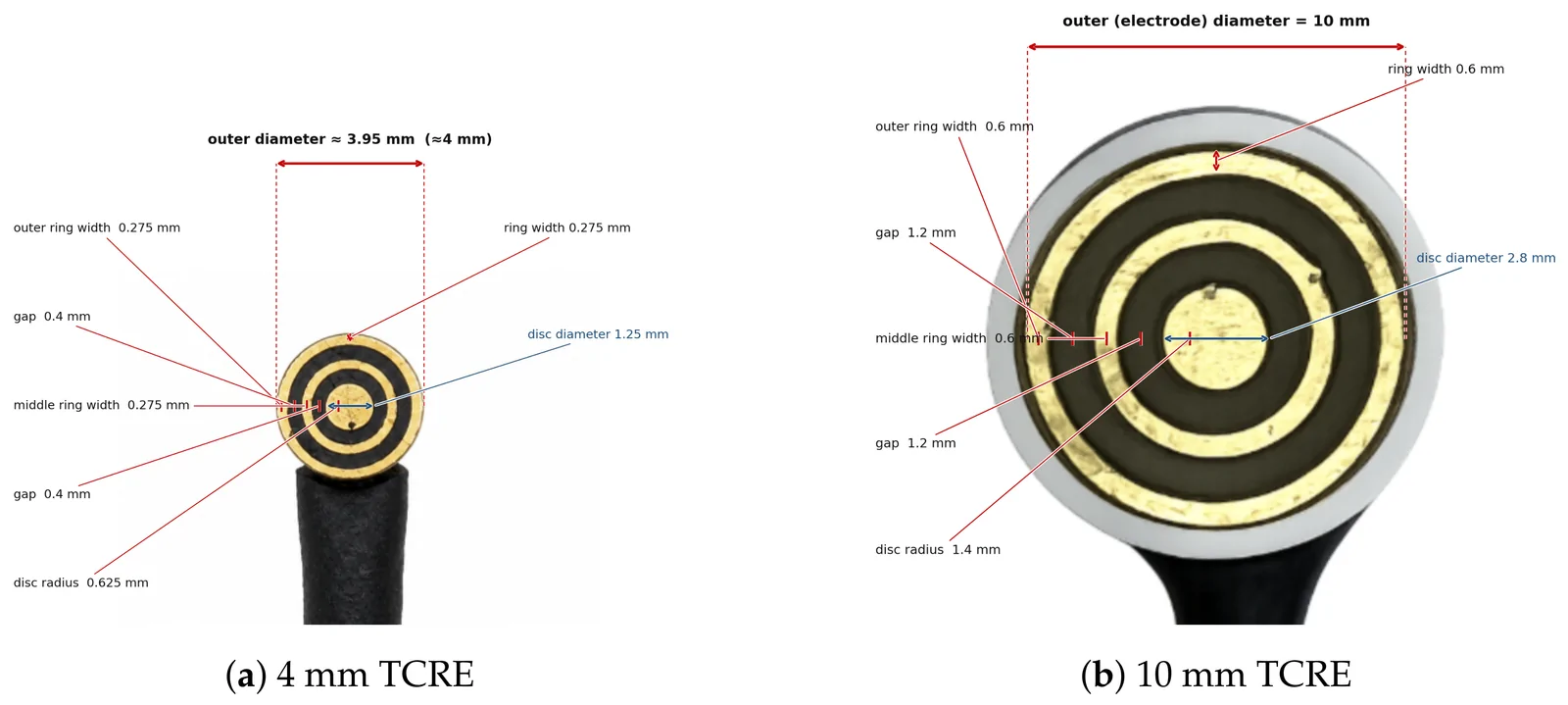
Tripolar concentric ring electrode (TCRE) configurations used in this study: (**a**) 4 mm and (**b**) 10 mm TCRE.

Figure 1 shows the two TCRE configurations used in this study. Each electrode has a central disc and two concentric rings about a common center. The surface Laplacian is estimated directly on the hardware [9]. The 4 mm and 10 mm electrodes have the same layout and number of rings. The two TCREs differ in size, with outer ring diameters of 10 mm and 4 mm. Table 1 lists the disc radii, ring radii, ring widths, and gaps of 4 mm and 10 mm TCRE sensors.

## 2. Materials and Methods

### 2.1. Dataset and Experimental Protocol

The dataset consisted of overt and covert speech EEG signals collected from 16 participants. This is the same dataset reported in our previous work [19]. In that study, conventional (emulated disc) and TCRE recordings were compared. The dataset is reanalyzed to compare the 4 mm and 10 mm TCREs. Therefore, some participant figures overlap with that study. Eight participants were recorded using 4 mm TCREs (P1–P4 and P10–P13). While the remaining eight participants were recorded using 10 mm TCREs (P5–P9 and P14–P16). Among those participants, 14 were right-handed and 2 were left-handed. The dataset included both monolingual and bilingual subjects. All participants were recorded at the Neural Rehabilitation Engineering Laboratory at the University of Rhode Island.

Each participant completed 200 trials (100 overt and 100 covert). The trials comprised four runs; each run consisted of either the overt or covert condition. The order of runs was counterbalanced across participants. Images were shown on a computer screen. Each image was displayed for 3500 ms and followed by a 2500 ms inter-stimulus interval during which the screen was gray to allow the participant to speak or imagine. EEG signals were recorded from the emulated disc and TCRE recordings. Each physical TCRE channel produced two outputs in the acquisition software: the outer ring provided an emulated disc signal, whereas the central disc, middle ring, and outer ring formed the TCRE, which estimates the surface Laplacian on the hardware, as shown in the above Equation (1).

**Figure 2:**
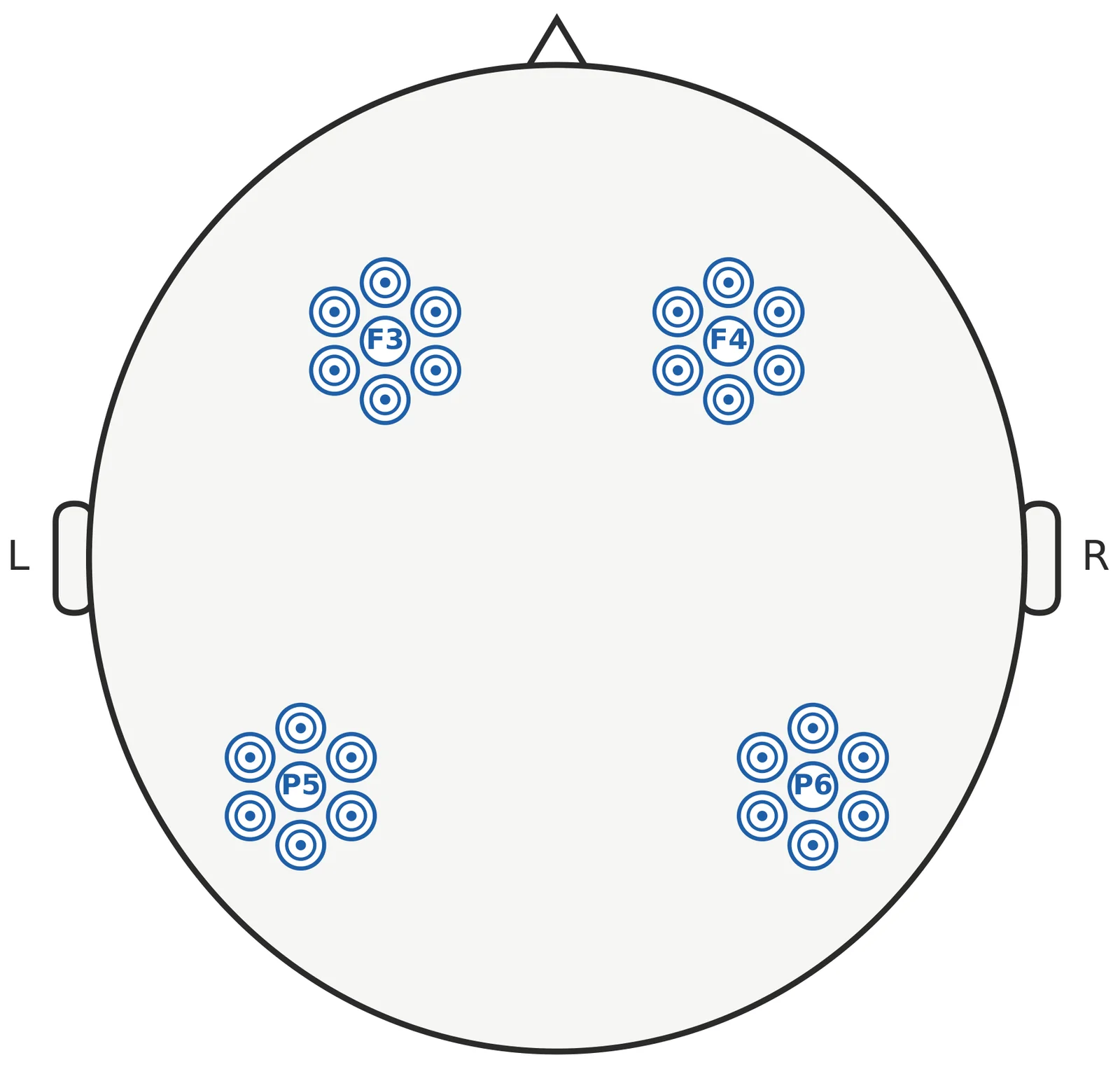
EEG sensor locations based on the 10–5 electrode montage system. TCRE arrays were placed at F3, F4, P5, and P6 for data collection.

The electrode positions followed the international 10–5 electrode placement system [20]. The array of TCREs was centered over cortical regions at F3, F4, P5, and P6, as shown in Figure 2. The number of TCREs per array varied across participants (5–8 per array). The total number of TCREs per participant ranged from 20 to 32. The green markers in Figure 2 indicate the individual TCRE position within each array. The EEG signals were sampled at 2500 Hz, and electrode-skin impedance was maintained below 10 kΩ during data acquisition. All participants were provided with a consent form prior to participation. The data collection procedures were approved by the Institutional Review Board (IRB) of the University of Rhode Island.

### 2.2. Signal Preprocessing

Prior to feature extraction, EEG recordings were band-pass filtered between 1 and 40 Hz using a zero-phase FIR filter with a Hamming window (MNE-Python 3.11.11 default). Frequencies below 1 Hz were removed to reduce baseline drift and low-frequency artifacts. While frequencies above 40 Hz were attenuated to suppress high-frequency noise and muscle artifacts. No independent component analysis (ICA) or artifact subspace reconstruction (ASR) was applied to this dataset. The band-pass filter attenuated the muscle activity. In addition, the EEG signals were segmented into epochs from −0.5 s to 2.5 s relative to stimulus onset. The interval from −0.5 s to 0 s served as the baseline period. The interval from 0 s to 2.5 s contained the overt or covert speech-related responses.

### 2.3. Spectral and Temporal Feature Analysis

Spectral and temporal analyses were performed to characterize the differences between the 4 mm and 10 mm EEG signals. Band-power features were extracted from four EEG frequency bands: theta (4–8 Hz), alpha (8–13 Hz), beta (13–30 Hz), and gamma (30–40 Hz). For each participant, band power values were computed and grouped according to the electrode type. Time–frequency representation (TFR) analysis was performed using the Morlet wavelet transform. Frequencies from 4 Hz to 40 Hz were analyzed. The baseline correction was performed using the pre-stimulus interval from −0.5 s to 0 s.

The magnitude of signal was quantified using the root-mean-square (RMS) value computed as

(2) $$RMS=\sqrt{\frac{1}{N}\sum_{i=1}^{N}x_{i}^{2}},$$

where $x_{i}$ represents the EEG amplitude and *N* denotes the number of samples within the analysis. RMS analysis was used to evaluate the differences in signal strength between EEG signals and speech conditions.

### 2.4. Classification Models

Participant-specific classification algorithms were employed to distinguish overt and covert speech state conditions. In addition, the classification was performed to analyze the differences between 4 mm and 10 mm TCRE EEG signals. The algorithms used in this study were Linear Discriminant Analysis (LDA), Support Vector Machine (SVM), and EEGNet.

The LDA and SVM classifiers were implemented in scikit-learn. In both classifiers, features were standardized to zero mean and unit variance using a StandardScaler within a scikit-learn pipeline. The LDA was implemented using the singular value decomposition solver. The LDA tolerance parameter was selected from $\left\{10^{-5},10^{-4},10^{-3},10^{-2},10^{-1}\right\}$ using five-fold cross-validation on the training and validation dataset. SVM models were evaluated using linear and radial basis function (RBF) kernels. The regularization parameter was selected C∈{0.1,1,10,100}. Hyperparameters were selected using five-fold cross-validation on the training dataset.

EEGNet, a compact convolutional neural network designed for EEG classification was also evaluated [21]. The input to the network was arranged as channels × time samples × 1. The model was trained using the Adam optimizer with a learning rate of 0.001 and binary cross-entropy loss. Dropout was selected from {0.25,0.50,0.75} using five-fold cross-validation on the training dataset. Once the model is trained, the best model based on hyperparameters was selected to refit the 80% of the dataset. The 20% dataset, which was unseen during 5-fold cross validation, was used to test the classification algorithms. Performance was evaluated using accuracy of test dataset.

### 2.5. Statistical Analysis

Non-parametric tests were used in this study due to the small sample size (n=8 per group). The significance level was set to p<0.05. The rank-biserial correlation (*r*) was used as the effect size. The Wilcoxon signed-rank test was used to compare the task-to-baseline RMS ratios of the emulated disc and TCRE signals. Both signals were recorded from the same participants. The Mann–Whitney U test was used to compare the 4 mm and 10 mm TCRE groups, because each group had different participants. This test was applied to both the RMS ratios and the classification accuracies. The classification accuracies were also compared with the 50% chance level [22]. For each electrode group, a one-sided Wilcoxon signed-rank test was applied to the accuracies of each classifier. For each participant, a one-sided binomial test was applied to the 40 test trials. Holm correction was used for multiple comparisons.

## 3. Results

### 3.1. Overt and Covert Temporal Responses

**Figure 3:**
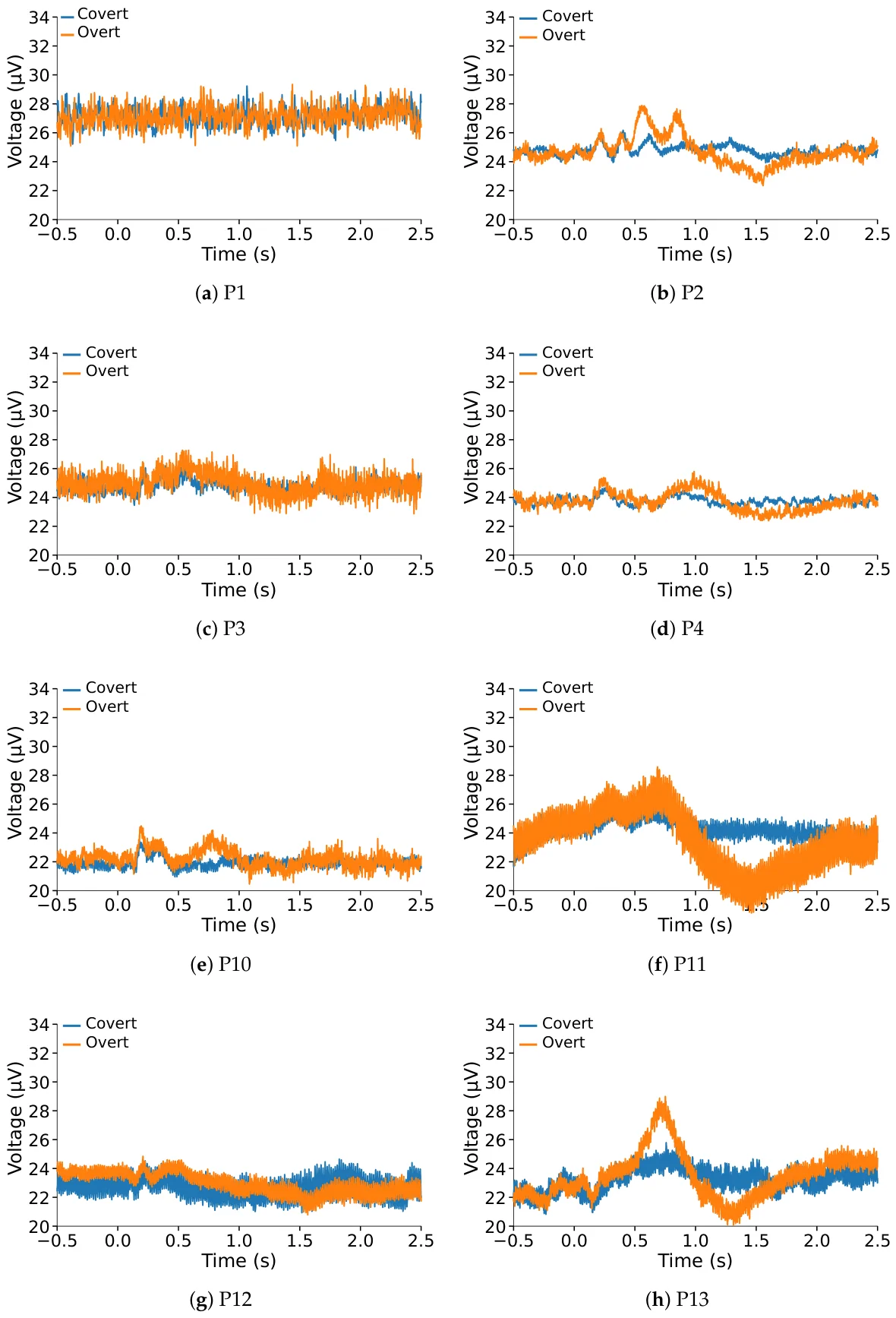
Participant-specific overt and covert EEG signals using emulated 4 mm disc electrodes. Subplots (**a**–**h**) correspond to individual participants (P1–P4, P10–P13). Each panel shows the averaged EEG amplitude (µV) over time (s) from −0.5 to 2.5 s; 0 s is the stimulus onset. Blue traces denote covert response, and orange traces denote overt response.

**Figure 4:**
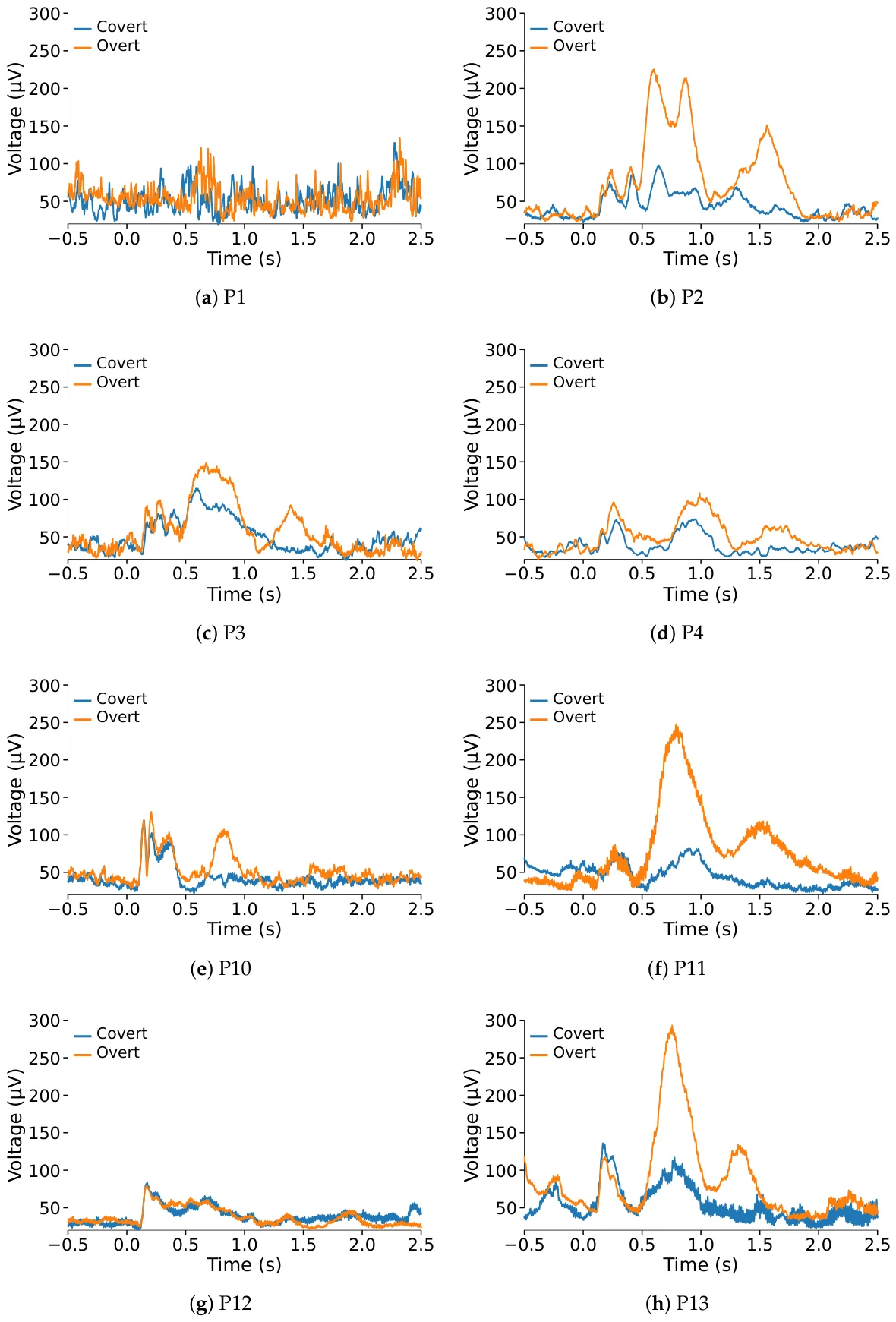
Participant-specific overt and covert EEG signals using 4 mm TCRE electrodes. Subplots (**a**–**h**) correspond to individual participants (P1–P4, P10–P13). Each panel shows the averaged EEG amplitude (µV) over time (s) from −0.5 to 2.5 s; 0 s is the stimulus onset. Blue traces denote covert response, and orange traces denote overt response.

Figure 3 and Figure 4 present the participant-wise averaged overt and covert speech responses. Each trace is the average across all trials of the corresponding condition for that participant. The inter-participant variability was observed across emulated and TCRE responses. This is the primary characteristic of EEG-based tasks [17]. The most prominent overt and covert difference was observed at 0.5 and 1.5 s after stimulus onset. The amplitude and temporal values varied across the participants. The disc EEG signals have a smaller amplitude compared to the TCRE signals, as shown in Figure 3. A clear difference in overt and covert EEG signals was observed in P11 using 4 mm TCRE signals, as shown in Figure 4. The 4 mm TCRE depicts a clear difference in P2, P11, and P13 with distinct overt peaks between 0.5 and 0.9 s after the stimulus onset.

**Figure 5:**
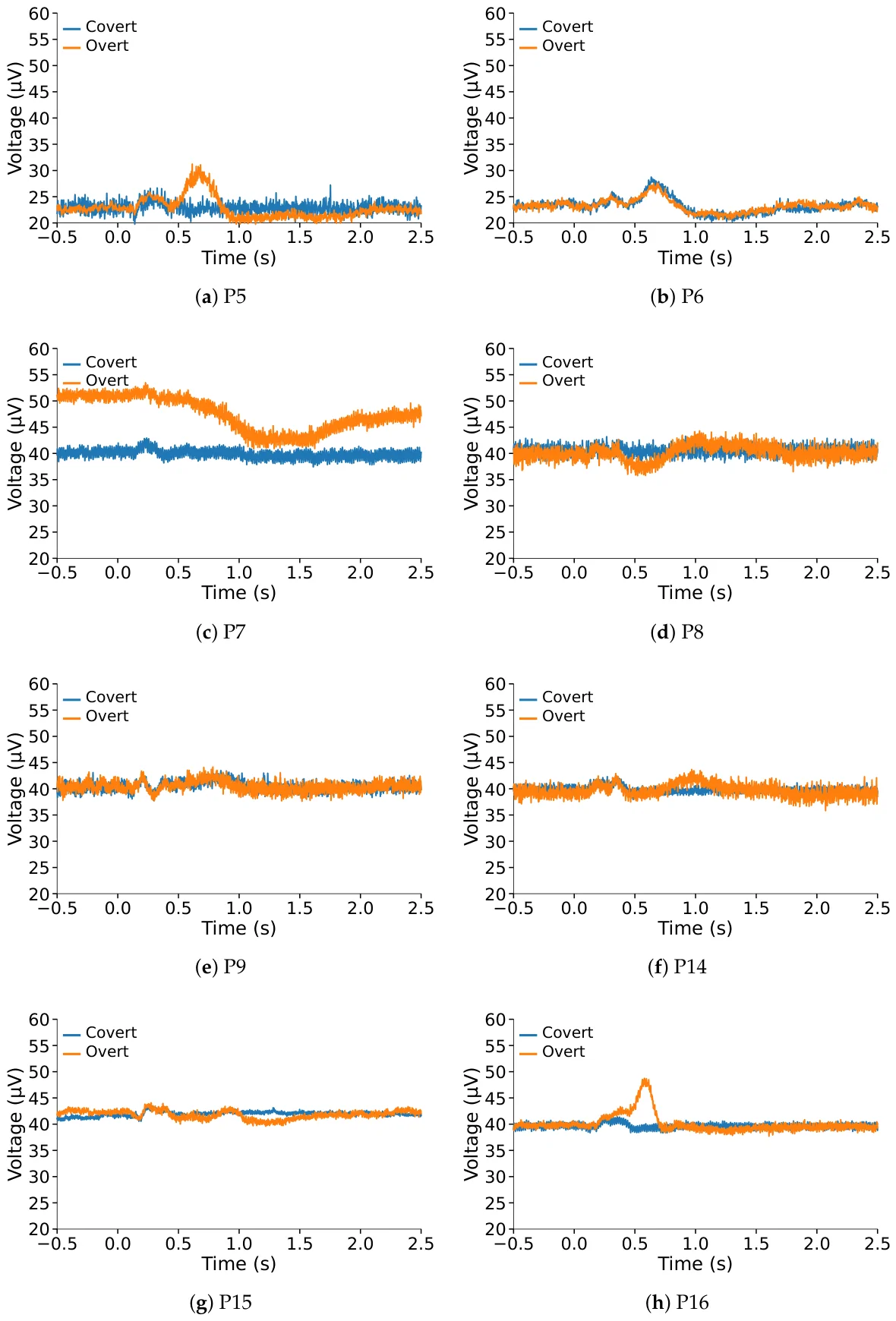
Participant-specific overt and covert EEG signals using emulated 10 mm disc electrodes. Subplots (**a**–**h**) correspond to individual participants (P5–P9, P14–P16). Each panel shows the averaged EEG amplitude (µV) over time (s) from −0.5 to 2.5 s; 0 s is the stimulus onset. Blue traces denote covert response, and orange traces denote overt response.

**Figure 6:**
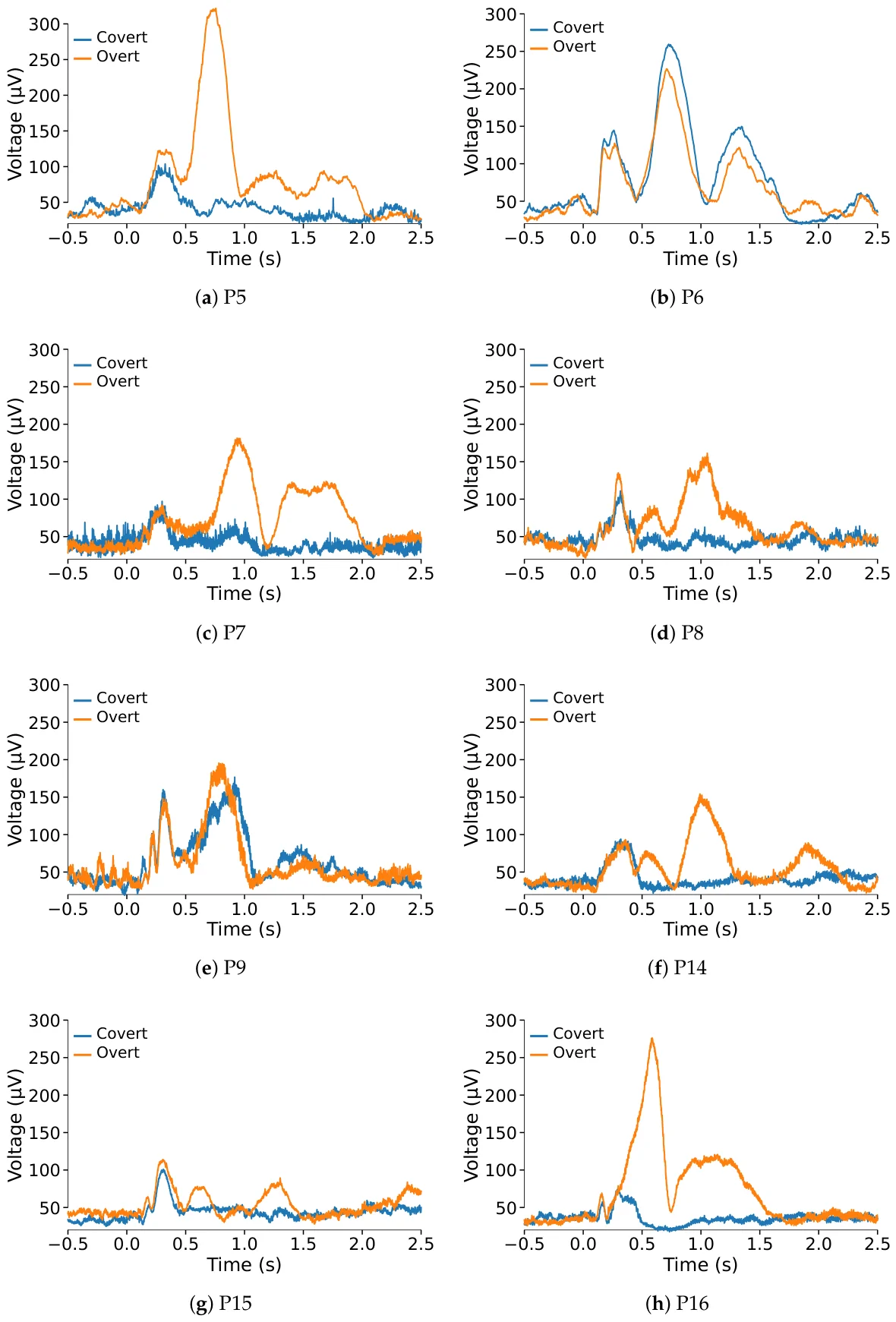
Participant-specific overt and covert EEG signals using 10 mm TCRE electrodes. Subplots (**a**–**h**) correspond to individual participants (P5–P9, P14–P16). Each panel shows the averaged EEG amplitude (µV) over time (s) from −0.5 to 2.5 s; 0 s is the stimulus onset. Blue traces denote covert response, and orange traces denote overt response.

Figure 5 and Figure 6 present overt and covert speech EEGs for emulated 10 mm disc and 10 mm TCRE signals. The overt has higher peaks compared to the covert signals in both emulated 10 mm disc and 10 mm TCRE signals. The P5, P6, P7, P14, and P16 have higher overt peaks and clear differences in the 10 mm TCRE.

### 3.2. Task-to-Baseline RMS Ratio

Task-to-baseline signal magnitude was computed by comparing the RMS amplitude during the task period with that of the pre-stimulus baseline period. The ratio in decibels was calculated as

(3) $${\mathrm{Ratio}}_{\mathrm{dB}}=20\log_{10}\left(\frac{{\mathrm{RMS}}_{\mathrm{task}}}{{\mathrm{RMS}}_{\mathrm{baseline}}}\right),$$

where ${\mathrm{RMS}}_{\mathrm{task}}$ and ${\mathrm{RMS}}_{\mathrm{baseline}}$ denote the RMS amplitudes computed during the task and baseline intervals, respectively. Positive values indicate that task-period EEG activity has a larger RMS amplitude than baseline activity, whereas negative values indicate lower task-period amplitudes relative to the baseline. Table 2 summarizes the task-to-baseline RMS ratios for overt and covert speech for all participants. For TCREs with a 4 mm diameter, the mean ratio was −0.397±1.105 dB during covert speech and 0.491±1.271 dB during overt speech. For the TCRE with a 10 mm diameter, the mean ratios were 0.338±1.755 dB and 1.843±1.204 dB, respectively. Overt speech produced a higher mean task-to-baseline ratio than covert speech for both TCRE and emulated disc electrodes.

Table 3 compares the task-to-baseline RMS ratios obtained from the emulated disc and TCREs with 4 mm and 10 mm EEG signals. For the three types of EEG signals, overt speech produced a higher mean ratio than covert speech. The overt ratio increased progressively from the emulated disc (0.103 dB) to the 4 mm TCRE (0.491 dB) and the 10 mm TCRE (1.843 dB). The covert ratios remained near zero for the disc, and 4 mm signals and were slightly positive for the 10 mm signals. Interparticipant variability was larger for the TCRE recordings than for the disc, as indicated in the higher standard deviations.

The larger ratios observed during overt speech are consistent with the greater signal magnitude visible in the time-domain responses, as plotted in Figure 3, Figure 4, Figure 5 and Figure 6. This indicates the sensorimotor activity associated with speech planning and articulation [13]. In addition, overt speech brings in facial muscle, vocalization, and movement-related activity [8]. Therefore, a higher task-to-baseline RMS ratio reflects both cortical and articulatory contributions rather than cortical activity alone.

### 3.3. Statistical Analysis of RMS Ratios

To further evaluate the effect of TCRE results on the task-to-baseline signal ratio, Wilcoxon signed-rank tests were performed to compare the RMS ratios obtained from the emulated disc and TCRE recordings. The Wilcoxon test was selected because the emulated disc and TCRE measurements were derived from the same participants, resulting in paired comparison. Non-parametric tests were used in this study because they do not assume a normal distribution and are also appropriate for the small sample size (*n* = 8 per group). The statistical results are summarized in Table 4. No statistically significant differences were observed between the disc and 4 mm TCRE signals for either covert or overt speech (p=0.313) and the effect sizes were medium (r=0.44). Similarly, no significant difference was observed between the disc and 10 mm TCRE signals during covert speech (p=0.945) with negligible effect size (r=0.06). However, a statistically significant difference was observed between the emulated disc and 10 mm TCRE signals during overt speech (p=0.008) with maximal effect size (r=1.00).

**Figure 7:**
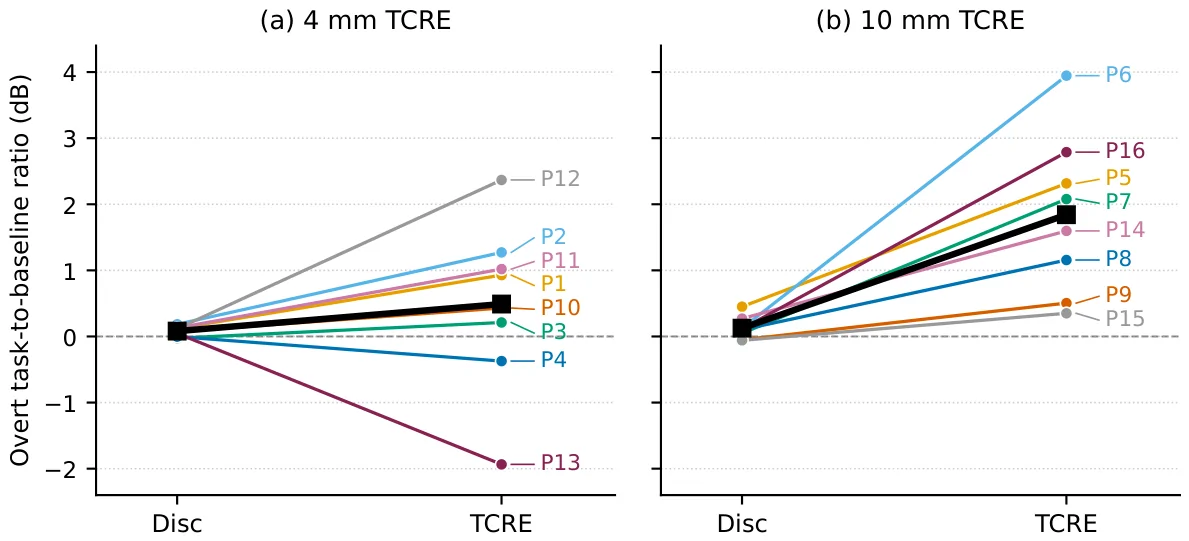
Comparison of overt-speech task-to-baseline RMS ratios obtained from TCRE recordings: (**a**) emulated disc with 4 mm TCRE and (**b**) emulated disc mm TCRE.

This finding is consistent with the participant-wise task-to-baseline RMS-ratio comparisons shown in Figure 7. The 10 mm TCRE showed an increased task-to-baseline RMS in each participant, whereas the 4 mm TCRE showed increased task-to-baseline RMS in all but two participants (P4 and P13). The 10 mm TCRE showed a task-to-baseline inter-participant variability among the participants.

Furthermore, Mann–Whitney U test was performed to compare RMS ratios between the two TCRE groups (4 mm and 10 mm). No significant differences were observed between the 4 mm and 10 mm TCREs for covert or overt ratios, as shown in Table 5. The effect sizes were 0.28 for covert and 0.56 for overt ratios. The lack of significance is due to the small dataset size in each group, rather than clear evidence that there is no effect.

### 3.4. Power Spectral Density

**Figure 8:**
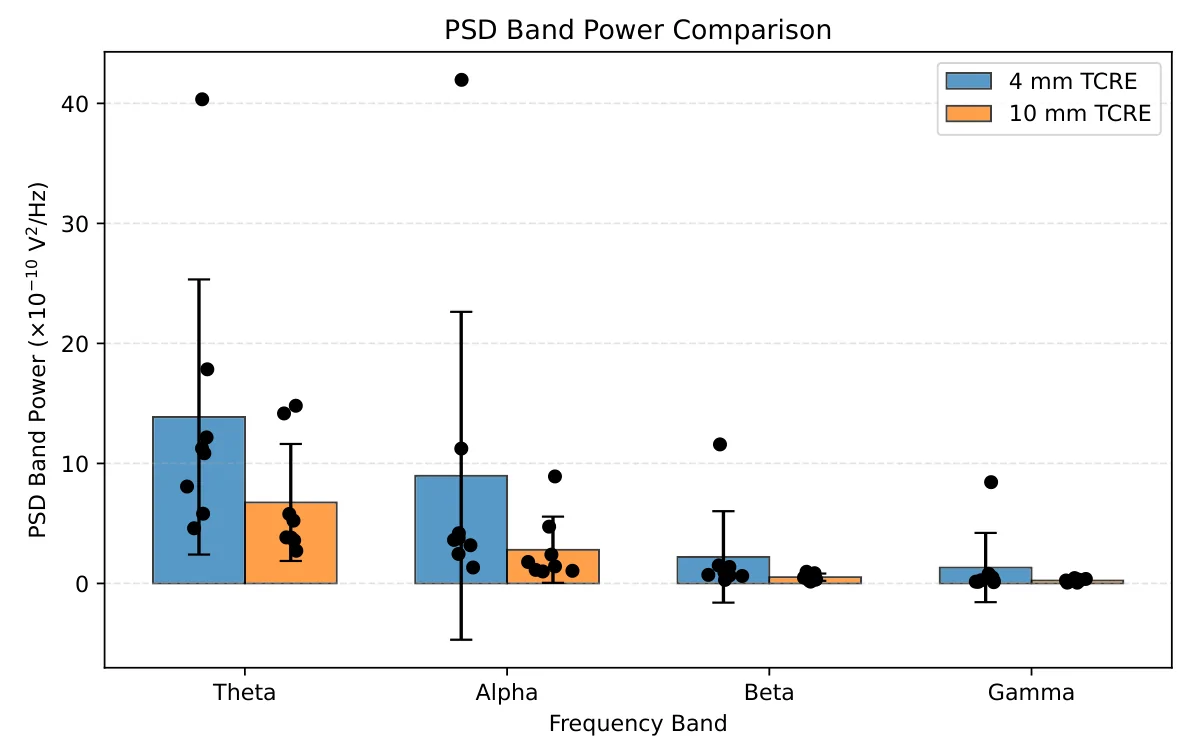
PSD band power comparison between the 4 mm and 10 mm TCRE groups across theta, alpha, beta, and gamma frequency bands. Bars represents the group mean, black points represent individual participants, and error bars represent the standard deviation across participants.

Figure 8 presents the Power Spectral Density (PSD) of theta, alpha, beta, and gamma frequency bands for the 4 mm and 10 mm TCRE groups. In both TCRE groups, band power was highest in theta and lower in the alpha, beta, and gamma bands. The 4 mm group exhibited higher mean power than the 10 mm group across all four frequency bands. The largest differences were observed in theta and alpha bands. In the theta and alpha bands, there was substantial inter-participant variability in the 4 mm TCREs. Some participants showed values higher than the remainder of the group. The beta and gamma had lower variability in both groups. These results illustrate the difference in variability between the two TCRE signals (4 mm and 10 mm).

### 3.5. Time-Frequency Representation Analysis

**Figure 9:**
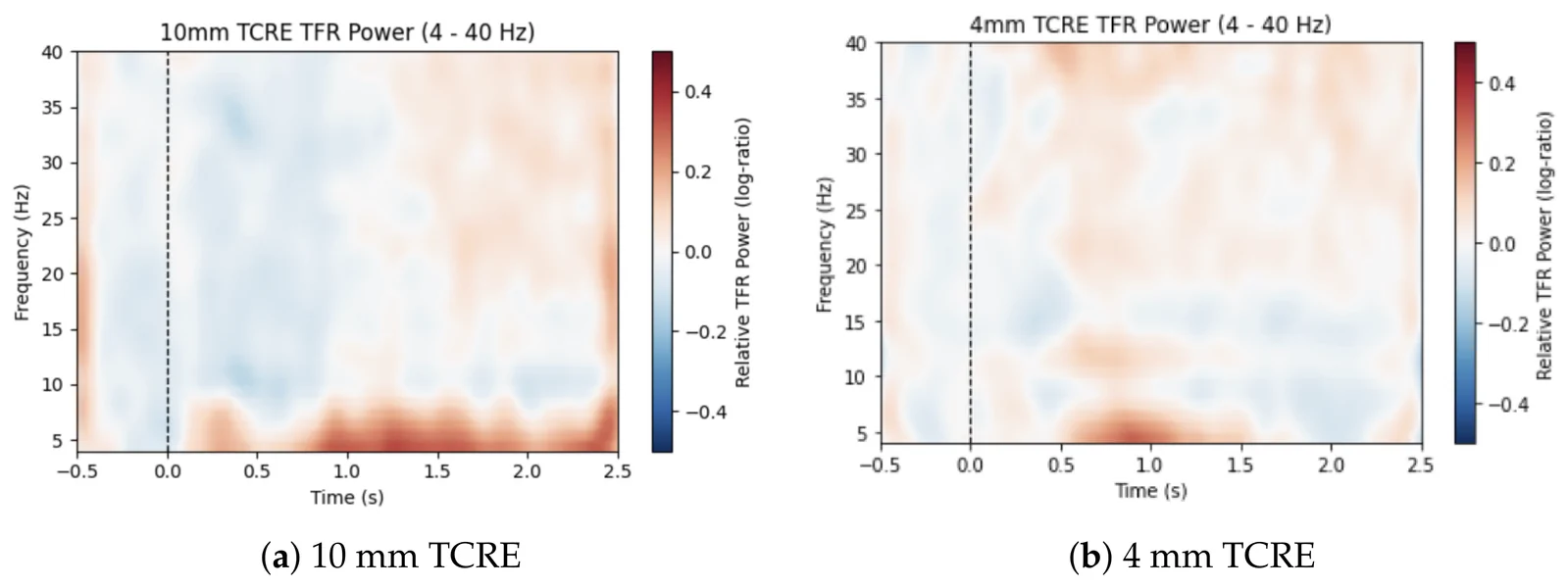
Time-frequency representations (TFRs) of EEG signals recorded using the (**a**) 10 mm TCRE and (**b**) 4 mm TCREs. The warm colors show the power increases and cool colors indicate power decreases.

Time-frequency representations (TFR) were analyzed using Morlet wavelets between 4 and 40 Hz. The spectral power was examined relative to the baseline period. Figure 9a and Figure 9b represent the TFR for TCREs 10 mm and 4 mm, respectively. Representative participants were selected to present the time-frequency responses observed in each TCRE group. Warm colors (red) indicate increases in power relative to the baseline period, whereas cool colors (blue) indicate relative power decreases.

Both representative TFRs showed post-stimulus power below 10 Hz. For 10 mm TCRE, the increased power is visible around 0.8 s and remained visible throughout the remaining epoch. For the 4 mm TCRE, an increase in power was shown between 0.6 and 1.5 s after the stimulus onset. The response reached its maximum power at 0.8–1.0 s. Then, it subsequently decreased in the remaining epoch. These representative plots suggest differences in the temporal and spectral behavior of the TCRE responses. However, they do not independently show a TCRE diameter effect. The plots were obtained from different participants and were influenced by inter-participant variability.

### 3.6. Classification Results

Table 6 summarizes the accuracies of participant-specific test data obtained using LDA, SVM, and EEGNet for overt and covert speech. For each participant, 20% of the trials were reserved for test data. The five-fold cross-validation was applied on the remaining 80% data set used for model selection and hyperparameter optimization. Each test dataset contained 40 trials. For the 4 mm TCRE group, the mean accuracies obtained using LDA, SVM, and EEGNet were 74.38±15.16%, 81.56±9.90%, and 79.38±11.78%, respectively. SVM achieved the highest mean accuracy and the lowest variability between participants. At the participant level, EEGNet provided the highest accuracy for P3 and P13, whereas SVM produced the highest accuracy for P1, P4, P10, and P11. All three models achieved the same accuracy for P2, while LDA performed best for P12. For the 10 mm TCRE group, the mean accuracies were 78.75±16.15%, 81.56±18.32%, and 88.75±18.18% for LDA, SVM, and EEGNet, respectively. EEGNet achieved the highest accuracy for six of the eight participants, including 100% accuracy for P7 and P8 on the 40-trial held-out test dataset. SVM achieved the highest accuracy for P16, whereas all three models performed at 45% for P6. These results show substantial variability among the participants. Among all 16 participants, EEGNet achieved the highest mean accuracy (84.06±15.57%), followed by SVM (81.56±14.23%) and LDA (76.56±15.30%). However, the performance of the classifiers varied among the participants. The high standard deviations indicate substantial variability between the participants. Therefore, the results suggest that the classifier performance depends on participant-specific EEG signals.

**Figure 10:**
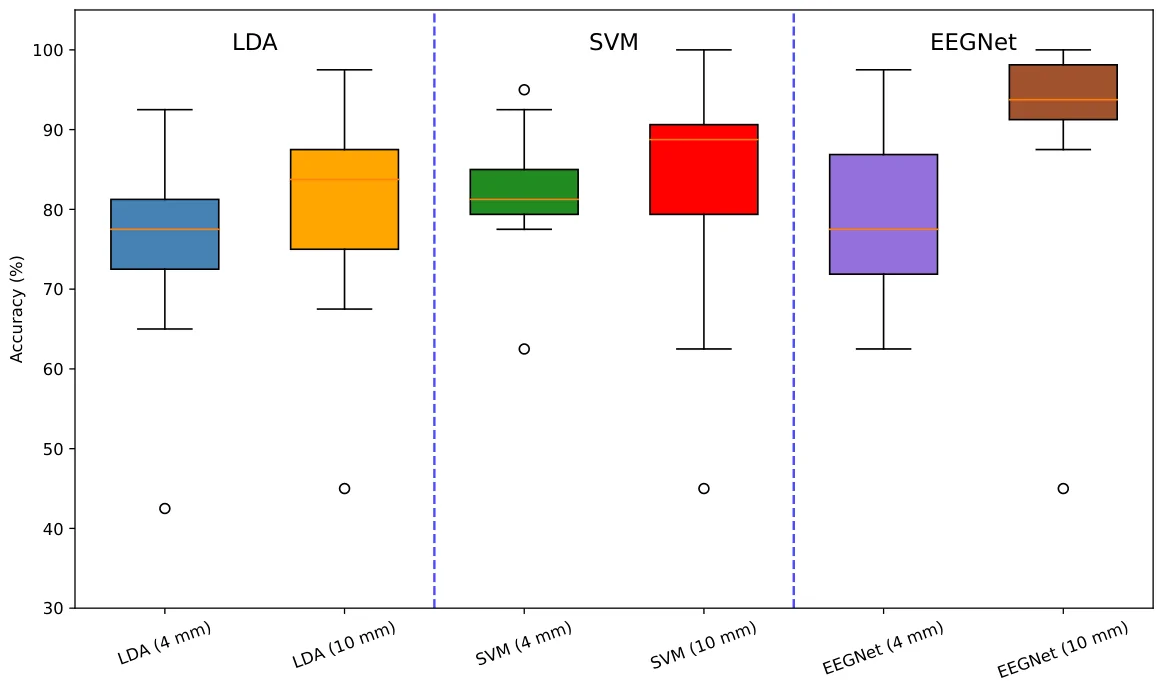
Distribution of participant-wise classification accuracies for 4 mm and 10 mm TCRE groups.

Figure 10 shows the distribution of participant-wise classification accuracies for the 4 mm and 10 mm TCRE groups. The 10 mm group showed higher median accuracies for all three classifiers, especially for EEGNet.

**Figure 11:**
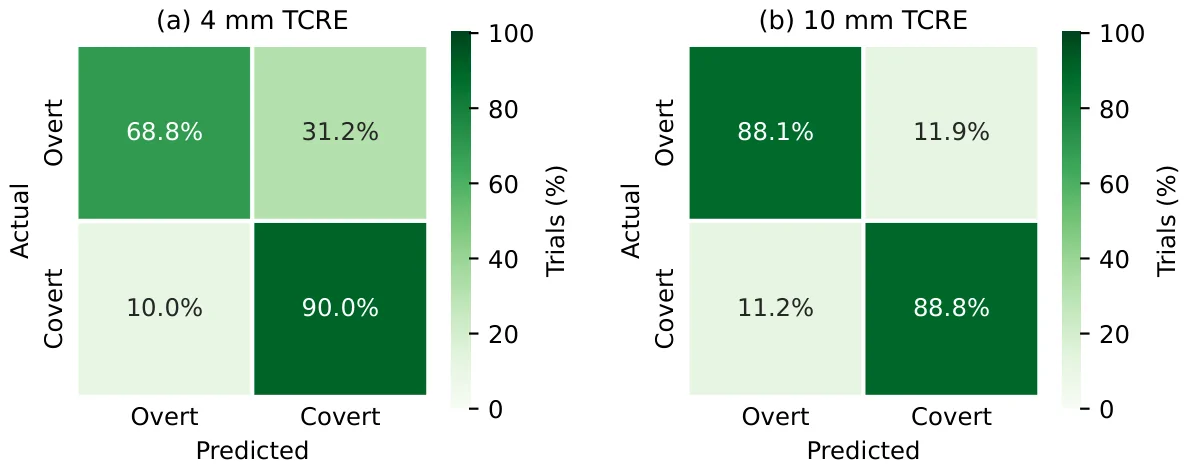
Aggregate EEGNet confusion matrices for overt and covert speech classification using (**a**) 4 mm and (**b**) 10 mm TCRE groups. Each cell represents the percentage of trials in each overt and covert class combined across the eight participants of each group.

Figure 11 presents the aggregate EEGNet confusion matrices for the 4 mm and 10 mm TCRE groups. In the 4 mm TCRE group covert speech was correctly classified with higher true positive (90.0%) than overt speech (68.8%). The result also indicates that a larger portion of overt trials were misclassified as covert (31.2%). In contrast, the 10 mm TCRE group showed a more balanced performance for overt and covert speech. The overt and covert showed true positives of 88.1% and 88.8%, respectively. These confusion matrices provide additional information by showing the distribution of classification errors between two speech states.

To further assess the class imbalance observed in the confusion matrices in Figure 11, precision, recall, and F1-score were calculated for EEGNet in Table 7. For the 4 mm group, precision (83.12%) was higher than recall (79.38%). The F1-score 78.31% was lower than the overall accuracy. This is consistent with the larger number of overt trials being misclassified as covert. For the 10 mm group, precision and recall were more similar (87.78% and 86.56%, respectively). This shows the more balanced confusion matrix observed in the 10 mm group.

### 3.7. Statistical Analysis of Classification Performance

The classification accuracies in Table 6 were compared with the 50% chance level. A one-sided Wilcoxon signed-rank test was applied to the accuracies of each classifier within each electrode group. A one-sided binomial test was applied to the 40 test trials of each participant with Holm correction. The results are presented in Table 8. All three classifiers performed above chance in both the 4 mm and 10 mm groups (p≤0.031). Six or seven of the eight participants in each group were above chance. Participant P6 from the 10 mm group remained at the chance level for all classifiers. These results show that both TCRE sizes supported overt and covert speech-state classification above chance.

The classification accuracies of the 4 mm and 10 mm groups were then compared using the Mann–Whitney U test. The results are presented in Table 9. No significant difference was found between the two groups for any classifier. LDA had a *p*-value of 0.343 and an effect size of 0.30. SVM had a *p*-value of 0.493 and an effect size of 0.22. EEGNet had a *p*-value of 0.073 and an effect size of 0.55. The effect sizes, together with the non-significant *p*-values, suggest limited statistical power due to the small sample size (n=8 per group).

Therefore, the results do not show that one TCRE diameter outperforms the other. Both electrode sizes achieved comparable classification performance in this dataset.

### 3.8. Limitations

Several limitations should be considered. First, 4 mm and 10 mm TCRE signals were collected from different participants. In particular, 4 mm TCREs data was collected from P1–P4 and P10–P13. The 10 mm TCREs data was recorded from P5–P9 and P14–P16. Therefore, the observed difference cannot be attributed to the TCRE diameter alone. Second, the dataset was small (n=16), which limits the accuracy and generalization of group-level comparisons. Third, the task distinguished speech states: overt or covert, rather than decoding words or linguistic content. Fourth, there is an electromyographic (EMG) contamination concern [23] during overt speech. Overt articulation engages facial and jaw muscles that generate myogenic activity. The band-pass filter was applied to remove the high-frequency muscle activities. The larger task-to-baseline RMS ratios and the classification performance observed during the overt speech may partly reflect articulatory and movement activities. In addition, TCRE recordings exhibit less muscle artifact than conventional EEG [24]. This reduces the muscle contribution during the overt speech. Fifth, the classification was performed using five-fold cross-validation within each participant but does not evaluate generalization to unseen subjects. Sixth, the disc recordings were emulated from the outer ring of the TCRE rather than obtained from a physical disc electrode. Prior work has validated the outer ring signal as an emulation of conventional disc electrode EEG [25]. Despite these limitations, the findings indicate that TCRE recordings and classification algorithms should be considered jointly for speech-state EEG analysis.

## 4. Conclusions

This study evaluated overt and covert speech EEG recorded with 4 mm and 10 mm TCREs using temporal, spectral, task-to-baseline RMS, TFR, and participant-specific classification algorithms. Both electrode types showed speech-state condition responses with substantial variability among the participants. Overt speech generally produced larger task-period amplitudes than covert speech. These differences include cortical, articulatory, facial-muscle, vocalization, and movement-related contributions [8]. The 10 mm TCRE produced a significantly higher overt response for task-to-baseline RMS ratios than the corresponding conventional disc recording, whereas 4 mm versus 10 mm group differences in RMS ratio and classification accuracy were not statistically significant. Among all participants, EEGNet showed the highest mean accuracy (84.06±15.57%), followed by SVM (81.56±14.23%) and LDA (76.56±15.30%). The results show the participant-wise TCRE-based overt and covert speech-state classification. Future work should use within-participant TCRE diameter comparisons and increase the dataset size to employ leave-one-subject-out (LOSO) validation to assess the generalization to unseen subjects. A recent study emphasizes the LOSO for practical BCI application [26]. In addition, the PSD should be extended to the high gamma band after the removal of EMG artifacts. A sliding window analysis could resolve the overt and covert separation at visual, planning, and articulation stages. The connectivity analysis between frontal and parietal regions could reveal how neural synchronization differs between overt and covert speech. The classification algorithms could be restricted to the pre-articulatory window to capture the cortical response of speech before motor artifacts. This would help for locked-in patients. Finally, the task can be extended from speech-state classification to word decoding.

## Figures and Tables

**Table 1:** Geometric dimensions of the two TCRE configurations.

Parameter	4 mm TCRE	10 mm TCRE
Central disc radius	0.625 mm	1.4 mm
Middle ring radius (inner–outer)	1.025–1.3 mm	2.6–3.2 mm
Outer ring radius (inner–outer)	1.7–1.975 mm	4.4–5.0 mm
Ring width	0.275 mm	0.6 mm
Inter-element gap	0.4 mm	1.2 mm
Outer (electrode) diameter	4 mm	10 mm

**Table 2:** Task-to-baseline RMS ratios for covert and overt across all participants.

Group	Participant	Covert Ratio (dB)	Overt Ratio (dB)
4 mm	P1	0.652	0.929
4 mm	P2	0.329	1.272
4 mm	P3	−1.031	0.212
4 mm	P4	−0.777	−0.372
4 mm	P10	−0.735	0.431
4 mm	P11	−1.268	1.020
4 mm	P12	1.469	2.368
4 mm	P13	−1.816	−1.935
4 mm TCRE Mean ± SD		**−0.397 ± 1.105**	**0.491 ± 1.271**
10 mm	P5	−0.451	2.316
10 mm	P6	4.209	3.946
10 mm	P7	−0.142	2.079
10 mm	P8	−0.327	1.157
10 mm	P9	1.041	0.505
10 mm	P14	−0.123	1.597
10 mm	P15	−1.800	0.351
10 mm	P16	0.295	2.789
10 mm TCRE Mean ± SD		0.338 ± 1.755	1.843 ± 1.204

**Table 3:** Comparison of task-to-baseline RMS ratios for emulated disc and TCRE signals.

Recording	Covert Ratio (dB)	Overt Ratio (dB)
Emulated Disc	−0.019±0.101	0.103±0.127
4 mm TCRE	−0.397±1.105	0.491±1.271
10 mm TCRE	0.338±1.755	1.843±1.204

**Table 4:** Statistical comparison of task-to-baseline RMS ratios between emulated disc and TCRE recordings using the Wilcoxon signed-rank test.

Comparison	*p*-Value	Rank-Biserial r
Emulated Disc vs. 4 mm TCRE (Covert ratio)	0.313	0.44
Emulated Disc vs. 4 mm TCRE (Overt ratio)	0.313	0.44
Emulated Disc vs. 10 mm TCRE (Covert ratio)	0.945	0.06
Emulated Disc vs. 10 mm TCRE (Overt ratio)	0.008	**1.00**

**Table 5:** Statistical comparison of task-to-baseline RMS ratios between the 4 mm and 10 mm TCRE groups using the Mann–Whitney U test.

Metric	*p*-Value	Rank-Biserial r
Covert ratio	0.382	0.28
Overt ratio	0.065	0.56

**Table 6:** Participant-specific test accuracies for overt and covert classification using LDA, SVM, and EEGNet.

Group	Participant	LDA (%)	SVM (%)	EEGNet (%)
4 mm	P1	42.5	77.5	72.5
4 mm	P2	80.0	80.0	80.0
4 mm	P3	75.0	82.5	85.0
4 mm	P4	80.0	82.5	75.0
4 mm	P10	65.0	80.0	62.5
4 mm	P11	92.5	95.0	92.5
4 mm	P12	75.0	62.5	70.0
4 mm	P13	85.0	92.5	97.5
4 mm Mean ± SD		74.38 ± 15.16	81.56 ± 9.90	79.38 ± 11.78
10 mm	P5	87.5	90.0	92.5
10 mm	P6	45.0	45.0	45.0
10 mm	P7	82.5	87.5	100.0
10 mm	P8	87.5	92.5	100.0
10 mm	P9	67.5	62.5	87.5
10 mm	P14	77.5	85.0	95.0
10 mm	P15	85.0	90.0	92.5
10 mm	P16	97.5	100.0	97.5
**10 mm Mean ± SD**		78.75 ± 16.15	81.56 ± 18.32	88.75 ± 18.18

**Table 7:** EEGNet precision, recall, and F1-score for the 4 mm and 10 mm TCRE groups.

Group	Precision (%)	Recall (%)	F1-Score (%)
4 mm TCRE	83.12±9.59	79.38±11.78	78.31±13.22
10 mm TCRE	87.78±18.34	86.56±18.71	86.28±19.03

**Table 8:** Statistical comparison of classification accuracy with the 50% chance level for the 4 mm and 10 mm TCRE groups. Group *p*-values were obtained using the one-sided Wilcoxon signed-rank test. Participants above chance were identified using the one-sided binomial test on 40 test trials. Holm correction was applied to both tests.

Classifier	4 mm Group *p*	4 mm Participants Above Chance	10 mm Group *p*	10 mm Participants Above Chance
LDA	0.031	6/8	0.031	6/8
SVM	0.023	7/8	0.031	6/8
EEGNet	0.023	7/8	0.031	7/8

**Table 9:** Statistical comparison of classification accuracy between the 4 mm and 10 mm TCRE groups using the Mann–Whitney U test.

Classifier	*p*-Value	Rank-Biserial *r*
LDA	0.343	0.30
SVM	0.493	0.22
EEGNet	0.073	0.55

## Data Availability

The data used in this study are not publicly available due to institutional restrictions. Data may be made available from the corresponding author with appropriate institutional approval.

## Abbreviations

The following abbreviations are used in this manuscript:

BCI	Brain–computer interface
EEG	Electroencephalography
IRB	Institutional Review Board
LDA	Linear discriminant analysis
PSD	Power spectral density
RMS	Root mean square
SVM	Support vector machine
TCRE	Tripolar concentric ring electrode
TFR	Time–frequency representation
